# 

**Published:** 1873-09

**Authors:** 


					TO HEADERS AND COIUIKSPONDENTSJ
Communications intended for publication, and exchange journals and pap<
should be addressed to the Editor of the American Journal of Deni
Science, 259 N. Eutaw St., Baltimore, Md. All letters relating to the busii
of the journal, or containing cash remittances, to be directed to Messrs. Snowi
& Cowman. Articles for publication should be received by the editor at
three weeks previous to the first day of the month on which the number in
it is, designed for tliem to appear, is issued.
SfigP'The American Journal of Dental Science will contain fifty pages
reading matter in each number, making a volume of not less than
Six Hundred pages
EXCLUSIVE OF ADVERTISEMENTS.
T 11 fi
AMERICAN JOURNAL
OF
DENTAL SCIENCE.
F. J. S. GORGAS, M. D., D. D, S., Editor.
The Journal is issued on the first of each month and contains not less
an fifty pages of reading matter in each number, exclusive of adver-
sements, paaking a volume of six hundred pages per annum.
In each number will be found Original Communications, Corres-
mdence, Selected Articles, Monthly Summary, Bibliographical No-
ees and Editorial Matter, and every effort will be exerted to render
le Journal worthy of the patronage of the Dental profession.
A generous cooperation is respectfully solicited from the members
7 the profession ; and as the Journal has now a printing office of its
wn which materially lessens the cost of its publication, it will be
sued at the reduced subscription price of
$2.50 For Annum in Advance.
Communications are respectfully solicited on all subjects pertain-
lg to the practice of dentistry, as well as reports of cases occurring
a dental' practice.
RATES OF ADVERTISING.
1 Page.
i "
i "
x ?
1 MO.
$15 00
10 00
7 00
5 00
2 MO.
'$22 50
15 00
ii oo
8 00
3 MO.
$30 00
20 00
15 00
11 00
6 MO.
$50 00
30 00
20 00-
15 00
12 MO.
$100 00
50 00
30 0C
20 00
All original communications designed for publication, 'works for
review, new instruments for notice, exchanges, &c., should be directed
to the editor, 259 N. Eutaw St., Baltimore, Md.
All advertisements and subscriptions should be addressed to
SNOWDEN & COWMAN,
Publishers of the Am. Journal of Dental Science.
86 W. Fayette St. Bet. Charles & Liberty Sts.,
BALTIMORE, Md.

				

